# Case Report: Severe wound formation following intratumoral tigilanol tiglate treatment resulting in limb amputation in a 10-year-old male dog

**DOI:** 10.3389/fvets.2026.1757258

**Published:** 2026-04-16

**Authors:** Liam Kitson, Jeffrey J. Runge, Mathew Shay

**Affiliations:** Department of Surgery, Guardian Veterinary Specialists, Brewster, New York, NY, United States

**Keywords:** canine (dog), case report, Stelfonta, systemic adverse effects, tigilanol tiglate

## Abstract

Tigilanol tiglate (TT; Stelfonta) is a novel intratumoral injection approved for the treatment of non-metastatic canine mast cell tumors (MCTs). Although the majority of reported adverse effects are localized and self-limiting, this case describes severe systemic and wound complications in a 10-year-old male Vizsla treated with a single 2 mL intratumoral injection for a subcutaneous mast cell tumor on the left lateral hock. Within 24 h, the dog developed vomiting, diarrhea, hypovolemic shock, and pancreatitis, requiring hospitalization and intensive supportive care. The cause of these systemic complications could not be definitively linked to Stelfonta, as multiple alternative diagnoses were also possible. Local tissue necrosis and wound infection subsequently progressed despite repeated surgical debridement, open wound management, and antibiotic therapy, culminating in distal limb necrosis and coxofemoral amputation 8 weeks post-injection. Histopathology confirmed proximal cicatrix formation, necrosuppurative inflammation, and fibrosis without residual neoplasia. This case highlights that while TT can be effective for local tumor control, clinicians must recognize the potential for rare but severe localized complications that may require surgical intervention.

## 1 Introduction

Mast cell tumors (MCTs) are the third most common type of tumor and the most common malignant skin tumor in dogs (1). They arise from bone marrow-derived mast cells, which normally participate in immune defense and tissue repair through the release of inflammatory mediators such as histamine, heparin, and proteases. These masses can show highly variable behavior, ranging from benign lesions to aggressive, metastatic disease. Prognosis is influenced by factors such as histologic grade, tumor location, and clinical stage, making thorough staging and careful management and treatment essential (2). Treatment of canine mast cell tumors typically involves surgical excision, with radiation therapy, chemotherapy, and tyrosine kinase inhibitors such as toceranib or masitinib used in cases with incomplete margins, high-grade disease, or metastasis.

Tigilanol tiglate (TT; Stelfonta) is a novel small-molecule agent labeled for use in veterinary medicine for the local treatment of non-metastatic cutaneous mast cell tumors located anywhere on the body and non-metastatic subcutaneous MCTs at or distal to the elbow or hock. The drug is administered via direct intratumoral injection, with the dosage based on the calculated tumor volume. It destroys tumors through oncolysis by activating protein kinase C (PKC). This activation leads to rapid immunogenic tumor cell death through vascular disruption and direct cytotoxicity, which promote further local and systemic antitumor immune responses (3, 4). This “tumor agnostic” method of action has prompted research into its effectiveness in other types of cancer and in other species, including two horses with non-resectable masses (5) and human cancer cell lines grafted onto mice (6). These studies have demonstrated the effectiveness of local chemoablation provided by TT, regardless of the tumor type.

The reported side effects of Stelfonta in canines are typically localized. These may include swelling, pain, erythema, and wound formation at the injection site due to tumor sloughing (10). These effects are typically self-limiting and directly related to the drug’s mode of action. The reported systemic adverse events are rare but usually related to mast cell degranulation, including lethargy, loss of appetite, vomiting, and even anaphylaxis. In humans, the intratumoral use of this medication for various types of tumors has reportedly caused adverse events such as respiratory arrest and sepsis (7). Concomitant medications (steroids and antihistamines) are recommended to minimize the risk of mast cell degranulation and its associated complications.

This case report describes the acute, progressive, and severe localized wound that ultimately led to hind limb amputation. The wound developed following a single intratumoral dose of Stelfonta (2 mg) administered to a 21.6 kg, 10-year-old intact male Vizsla. The injection targeted a subcutaneous MCT on the left lateral hock. Despite repeated attempts at management, the condition necessitated hindlimb amputation.

## 2 Case description

A 10-year-old intact male Vizsla was referred to the oncology service at a privately owned veterinary specialty hospital for a subcutaneous haired MCT on the lateral aspect of the left tarsus. The mass was originally diagnosed via fine-needle aspiration (FNA) on 26 February 2025 by the primary veterinarian, who started the patient on oral diphenhydramine (2.2 mg/kg, twice daily), omeprazole (1 mg/kg, twice daily), and prednisone (1 mg/kg, twice daily for 10 days, then once daily). At the oncology consultation, repeat FNA confirmed a low cytologic grade MCT. Staging included three-view thoracic radiographs, abdominal ultrasound, CBC, serum biochemistry, urinalysis, and a Nu. Q® cancer screen. No evidence of metastasis was identified. Mild neutropenia (3.38 K/μL) and a subjectively coarse hepatic echotexture were noted but considered clinically insignificant. The tumor met the labeled inclusion criteria for intratumoral tigilanol tiglate treatment (non-metastatic, low-grade subcutaneous MCT distal to the hock). The patient was then scheduled to return in 8 days (day 0) for Stelfonta treatment and was discharged on oral famotidine (0.5 mg/kg, twice daily), diphenhydramine (2 mg/kg, twice daily), prednisone (0.5 mg/kg, twice daily) and gabapentin (14 mg/kg, to be given on the morning of treatment).

On 20 May 2025 (Day 0), an IV catheter was placed, and the patient was sedated with 5 μg/kg of Dexdomitor and 0.2 mg/kg of Torbugesic (Zoetis). The tumor volume was estimated at 4cm^3^, and an intratumoral injection (2 mL) of Stelfonta was administered into the MCT site at the proximolateral aspect of the left tarsus. Sedation was then reversed with 0.2 mL of Antisedan (Zoetis) IM. The patient recovered uneventfully from sedation and was discharged with instructions to continue famotidine for 8 more days, adjust diphenhydramine to every 8 h for 4 days, then every 12 h for 4 days. Prednisone was prescribed on a tapering schedule: 10 mg every 12 h for 5 days, then once daily for 7 days, followed by a 1/2 tablet once daily for 7 days, and finally a 1/2 tablet every other day for three doses.

Approximately 41 h post-injection (Day 2), the owner reported acute diarrhea and vomiting, without dietary indiscretion. The owner was advised to bring the dog to the emergency service, and the patient presented 8 h later. On examination, the dog was quiet to dull, dehydrated, and hypotensive, with pale mucous membranes, tachycardia (230 bpm), prolonged capillary refill time (>2 s), weak femoral pulses, and a systolic blood pressure of 100 mmHg (Doppler). Point-of-care testing revealed a lactate of 8.3 mmol/*L. spec* cPL was 1273.6 ng/mL. CBC demonstrated leukopenia (WBC 3.2 K/μL), and biochemistry showed mild ALP elevation and mild hyperglycemia. Abdominal ultrasound demonstrated mildly hyperechoic peripancreatic fat. A diagnosis of hypovolemic shock with clinicopathologic and ultrasonographic findings consistent with pancreatitis was made.

The patient received two 20 mL/kg IV boluses of Ringer’s lactate solution, which resolved the tachycardia. He was subsequently hospitalized under internal medicine for supportive care, including IV LRS (90 mL/kg/day, adjusted to maintain normotension), maropitant (1 mg/kg IV once daily), methadone (0.2 mg/kg IV every 6 h), famotidine (0.5 mg/kg IV twice daily), dexamethasone SP (0.1 mg/kg IV once daily), ampicillin/sulbactam (30 mg/kg IV every 8 h), and diphenhydramine (2 mg/kg IM twice daily), with close monitoring. Vomiting resolved, and the fluid deficit was corrected within 24 h. Appetite returned by day 5 post-injection (Day of discharge), and normal, formed stool was observed at home on day 7.

The patient’s one-week oncology recheck coincided with his fifth and final day of hospitalization, during which the oncology team determined that the treatment site was appropriately debriding and progressing. The patient was discharged with instructions to continue previously prescribed medications. In addition, the patient was dispensed Proviable (Nutramax) to be given daily, along with as-needed oral medications for vomiting and diarrhea: Maropitant (2 mg/kg once daily), metoclopramide (0.25 mg/kg), and Canalevia-CA1 (one tablet twice daily for 3 days) (Table 1).

**Table 1:** Timeline of disease progression and treatments.

Date/Day post-injection	Clinical event
02/26/25	Initial diagnosis of subcutaneous MCT by the primary veterinarian; started prednisone, diphenhydramine, and omeprazole.
05/12/25	Oncology consultation; low-grade MCT confirmed; staging unremarkable; Stelfonta treatment scheduled.
05/20 (Day 0)	Intratumoral injection of tigilanol tiglate (2 mL).
05/21–05/25 (Days 1–5)	Onset of vomiting, diarrhea, and hypovolemic shock; hospitalization; pancreatitis diagnosed.
Day 9	Severe circumferential necrosis identified, with mild pyrexia and leukocytosis; wound cultures positive for *E. coli*, *Enterobacter*, and *Klebsiella* spp.; enrofloxacin instituted; beginning of hospitalization.
Day 13	First surgical debridement; culture and sensitivity results guided discontinuation of enrofloxacin and initiation of meropenem and clindamycin.
Day 15	Second surgical debridement.
Day 26	Third surgical debridement.
Day 36	Fourth surgical debridement; cicatrix removal; releasing incision.
Day 53	Discharged after 44 days of hospitalization.
Day 59	Declining QOL; coxofemoral amputation performed for non-salvageable limb.
Post-operative	Uneventful recovery; normal ambulation on three legs within 1 week; return to normal appetite and energy within 1 month.

## 3 Diagnostics, therapeutic interventions, follow-up, and outcomes

### 3.1 Initial wound formation

**Figure 1:**
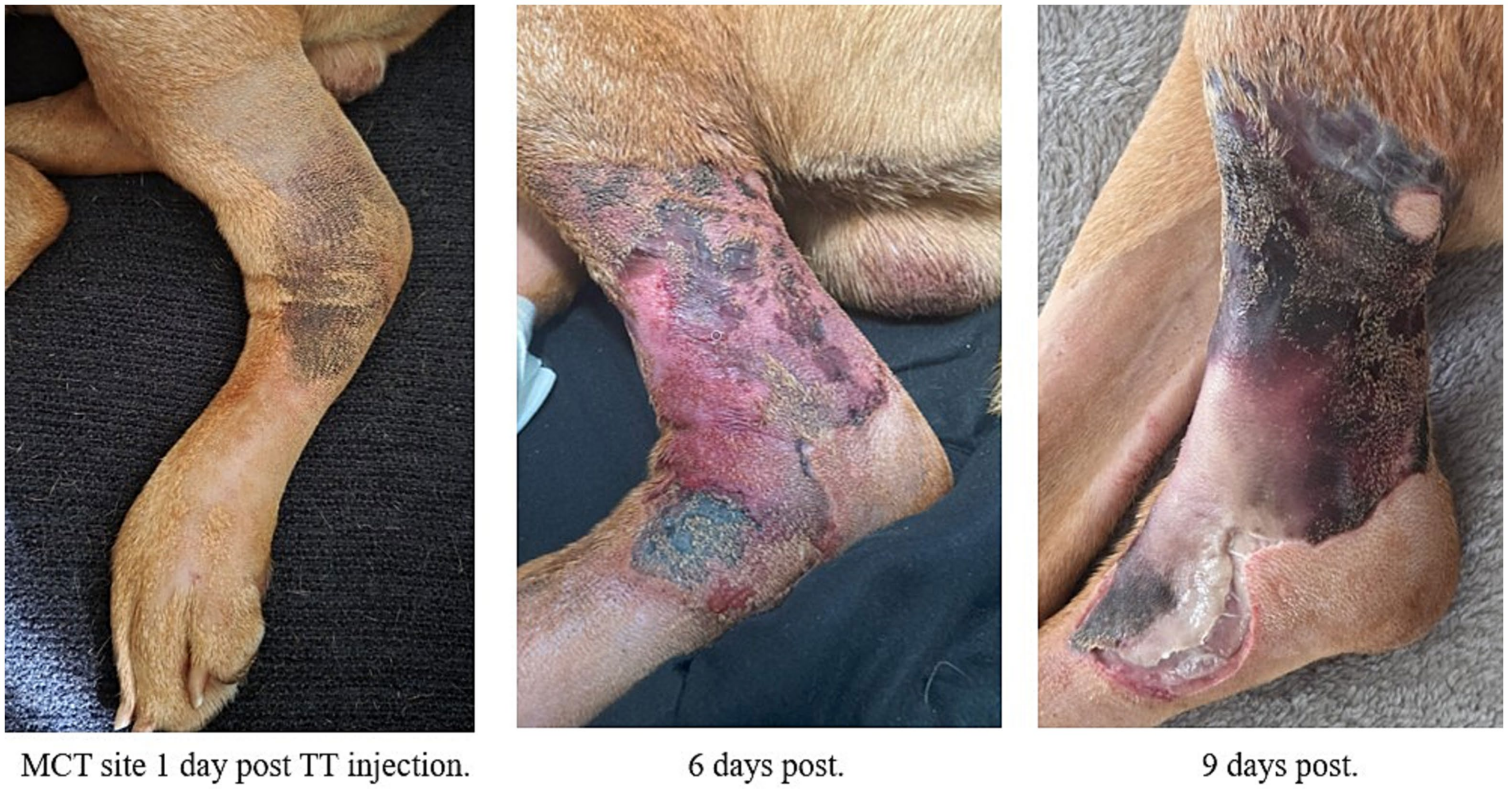
Progression of the left hindlimb MCT site wound at day 1 post-injection (left), day 6 (center), and day 9 (right).

Within 24 h of intratumoral injection, localized erythema and edema developed at the injection site on the proximolateral aspect of the left hock. A total of 6 days after injection, the area developed severe circumferential edema and erythema around the injection site. No discharge or exposed deep layers were noted at this time (Figure 1).

On day 9, the patient returned to the emergency service due to owner-reported non-weight-bearing lameness of the affected limb, as well as chewing and licking at the site. Physical examination revealed a wound stable in size (7×10 cm, with circumferential involvement of 270° around the tarsus) but with marked tissue necrosis and sloughing (Figure 1). Circumferential eschars were noted along wound edges. Odorous, white, purulent discharge was observed oozing from the lateral side of the distal wound edges, where exposed muscle was also observed. The samples were submitted for aerobic culture and sensitivity. These cultures grew *E. coli*, *Enterobacter* sp., and *Klebsiella aerogenes*. While the *E. coli* and *Klebsiella* isolates were broadly susceptible to different antibiotic classes, it should be noted that the *Enterobacter* isolate was carbapenem-resistant (CRE) and reportedly susceptible only to aminoglycosides. Antibiotic therapy was adjusted accordingly once the culture results became available.

The patient was once again hospitalized under the care of the internal medicine service. Empiric antibiotic therapy was started with IV ampicillin/sulbactam (30 mg/kg IV every 8 h) and cefazolin (22 mg/kg IV twice daily), along with IV crystalloids (LRS, 90 mL/kg/day, adjusted to maintain normotension). Previously prescribed supportive medications were continued in injectable form: Maropitant (1 mg/kg IV q24h), methadone (0.2 mg/kg IV q4–6h), famotidine (0.5 mg/kg IV q12h), and dexamethasone SP (0.1 mg/kg IV once). Topical manuka honey was applied twice daily.

### 3.2 Wound deterioration and infection (day 8–13 post-injection)

Between days 8 and 10, the wound area expanded to 8 × 10 cm, encompassing approximately 270° of the distal limb circumference with deep dermal and subcutaneous necrosis. Edema of the distal extremity increased, and the patient became non-weight-bearing. Purulent exudate with a foul odor was also observed. Cytology of the wound exudate revealed mixed cocci and rods; aerobic culture grew *E. coli*, *Enterobacter* sp., and *Klebsiella aerogenes*, with the latter two resistant to *β*-lactams but susceptible to aminoglycosides. Broad-spectrum ampicillin/sulbactam (30 mg/kg IV q8h) was continued pending sensitivity results. Once sensitivity results were available, ampicillin/sulbactam was discontinued, and enrofloxacin (9 mg/kg PO twice daily) was instituted. Systemic signs included mild pyrexia (103.8 °F) and leukocytosis (WBC = 26 × 10^9^/L). The decision was made to proceed with surgical debridement.

### 3.3 First surgical debridement

**Figure 2:**
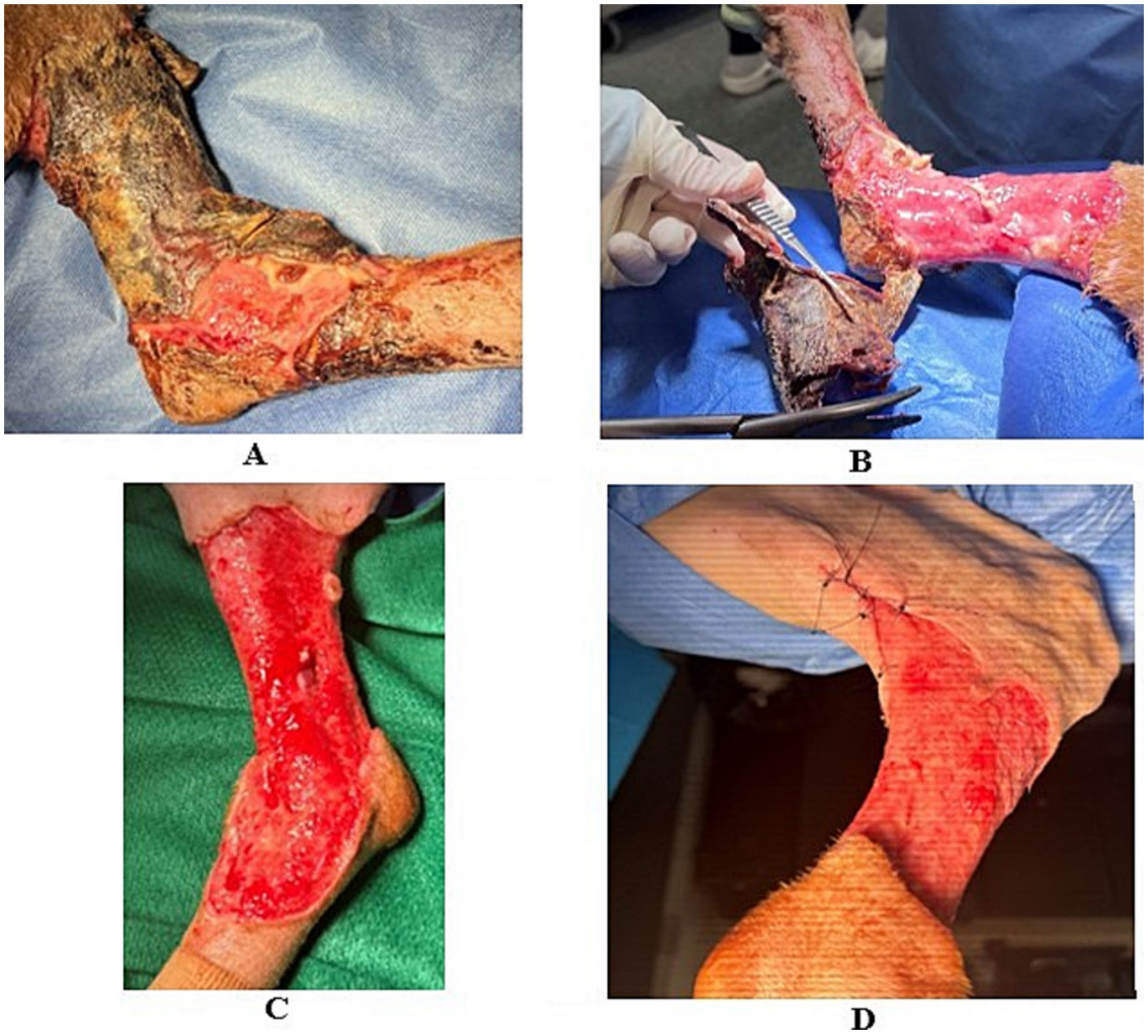
Day 13: Stages of the first surgical debridement. Before debridement **(A)**, removal of eschar during debridement **(B)**, after lavage **(C)**, and a partially closed caudoproximal aspect of the wound **(D)**.

The first debridement (Figure 2) was performed on day 13 post-injection. The large circumferential eschar (7 × 11 cm), surrounding the wound from the mid-tibia to the metatarsals, was removed. All necrotic tissue was excised using sharp debridement; the wound bed was mechanically debrided with sterile gauze and a #10 blade until capillary bleeding was observed. The wound was lavaged with 3 L of sterile saline using a SurgiLav system. Partial closure of the caudoproximal quadrant was achieved with 2–0 nylon tacking sutures, followed by application of honey-impregnated gauze for autolytic debridement. The limb was supported with a lightly padded Robert Jones bandage. Samples were submitted for repeat aerobic culture and sensitivity, which grew *Enterobacter* sp. and *Klebsiella pneumoniae*. The *Enterobacter* isolate was consistent with the previous culture (CRE, susceptible only to aminoglycosides), while the *Klebsiella* isolate was susceptible to marbofloxacin, amikacin, and carbapenem. Therapy was adjusted to initiate meropenem (8 mg/kg SQ twice daily) and clindamycin (15 mg/kg PO twice daily). Pain was managed with methadone (0.2 mg/kg IV q6h) and gabapentin (10 mg/kg PO q8h). The patient remained hospitalized for monitoring and analgesia, with bandages changed every 12 h, and partial granulation was expected once the necrotic tissue demarcated.

### 3.4 Ongoing open wound management and repeat debridement

Open wound management and supportive care were continued during hospitalization, with three additional rounds of surgical debridement performed.

*Day 15* (Second debridement): Dried, crusted discharge and debris were present on the superficial surface of the wound. The wound edges were healthy and indicated progressive contraction. The wound extended 360° around the tarsus. After cleaning with a chlorohexidine scrub, mild proliferative granulation tissue was noted across the entire wound, which still measured approximately 7×11 cm; a central fibrinous plaque persisted. Overall, the leg edema was decreasing. The wound surface was mechanically debrided for the second time using a combination of sterile gauze and a #20 blade until capillary bleeding was achieved. Debridement continued until the wound bed appeared cleaner and healthier. The wound bed was then lavaged, and the edges were reassessed.

**Figure 3:**
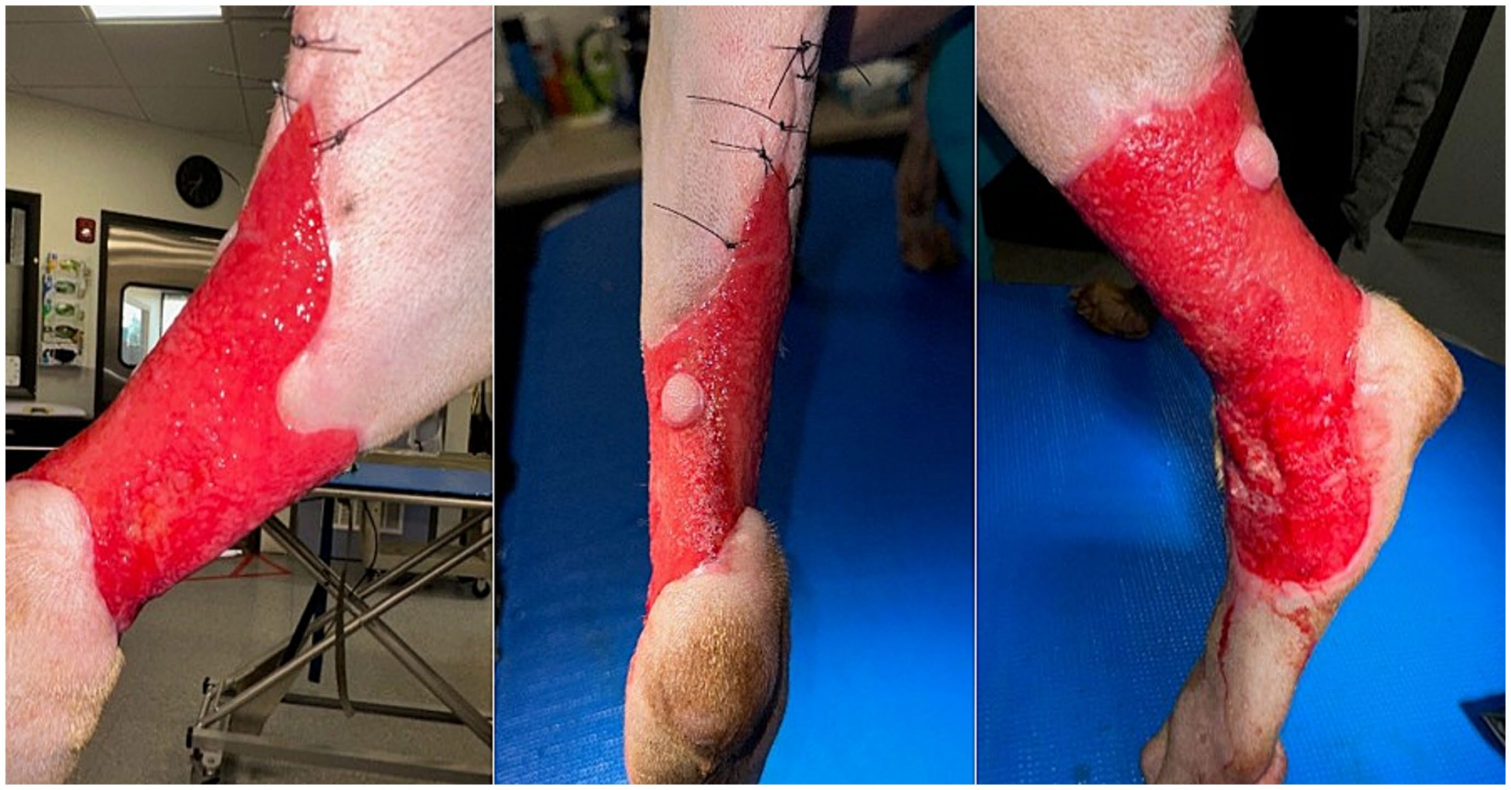
Day 26: Caudomedial view of the wound after debridement (left). Caudal view (center). Lateral view (right).

*Day 26* (Figure 3): Persistent exudate and delayed granulation tissue prompted the third surgical debridement, during which biofilms and debris were noted on the superficial surface of the wound. At this time, the wound measured approximately 6×10 cm and still extended 360° around the tarsus. The wound was once again debrided using a combination of sterile gauze and a #20 blade. Debridement continued until the wound bed appeared healthier with capillary bleeding exposed.

A shave biopsy was taken from the superficial layer of the craniolateral aspect of the wound bed, just proximal to the hock (the previous MCT site), to ensure no recurrence of neoplasia. Histopathology of the biopsy revealed fibrin and fibroplasia with a mixed lymphoplasmacytic and neutrophilic infiltrate. Repeat culture and sensitivity performed at this debridement grew *Staphylococcus pseudintermedius*, which was sensitive only to aminoglycosides, amphenicols, and mupirocin. Chloramphenicol was added (50 mg/kg per os every 8 h) and continued for the remainder of the hospitalization.

**Figure 4:**
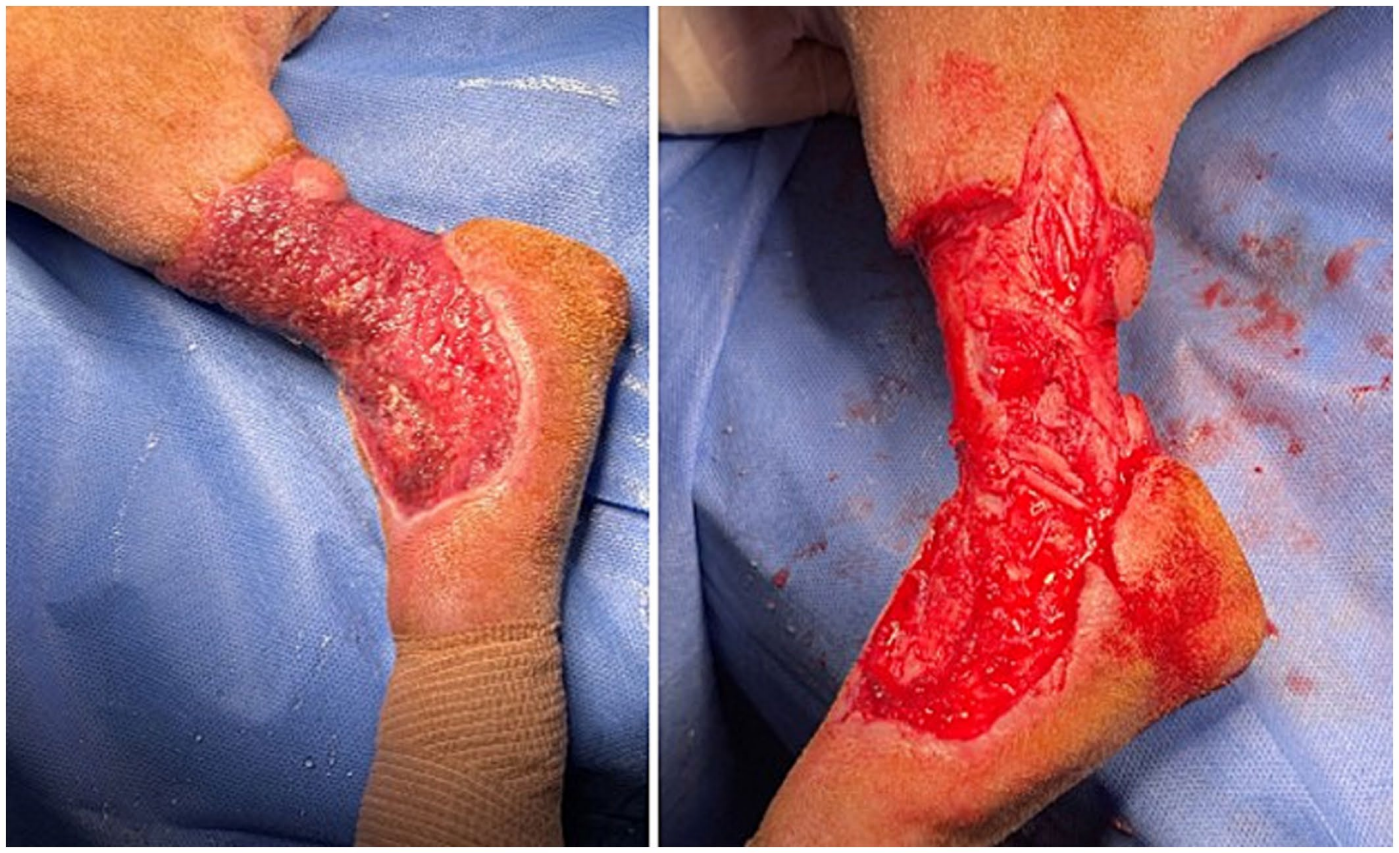
Left hindlimb before (left) and after (right) the fourth and final debridement, including cicatrix removal and releasing incision.

*Day 36*: The fourth and final debridement (Figure 4) was performed in the same manner as previous sessions, except it included removal of a significant circumferential cicatrix extending ~1 cm deep at the proximal aspect of the wound bed and the creation of a 2.5 cm linear releasing incision running distal to proximal along the lateral aspect of the proximal wound edge. After this, the wound area was reduced to 6×8 cm, with improved drainage and minimal edema.

### 3.5 Late course and decision for amputation

On day 53 post-injection (after 44 days of hospitalization), the patient was discharged with instructions to continue antibiotics (Chloramphenicol 50 mg/kg per os every 8 h), trazodone (5 mg/kg per os every 8–12 h), OWM, and physical therapy at home. Despite four aggressive wound debridements, broad-spectrum antibiotics, and supportive care, epithelization failed, and the patient continued to experience persistent distal edema and non-weight-bearing lameness. A total of 6 days after discharge, after seeking second opinions from other surgical specialists, the owner determined that, despite previous treatments, the condition had not improved. Therefore, the patient’s quality of life was greatly compromised, and due to the chronic infection risk and poor perfusion, amputation was elected on day 59 post-injection.

On presentation for amputation, the limb was diffusely edematous, with raised circumferential wound margins and induration extending circumferentially at the proximal edge. At coxofemoral disarticulation, diffuse necrosuppurative inflammation and fibrosis were confirmed grossly. The procedure was uneventful, and postoperative analgesia included an epidural morphine/bupivacaine combination and oral carprofen (2 mg/kg PO q12h). Histopathology of the limb confirmed necrosuppurative inflammation and fibrosis, with no residual mast cells or bacteria detected.

### 3.6 Outcomes and follow-up

The patient recovered uneventfully from amputation, resumed ambulation within 1 week, and demonstrated normal appetite and energy by 1 month. No further systemic illness or wound complications were reported. At the time of writing, the patient was doing well, with gradual improvements in comfort and mobility at home and no lingering adverse effects.

## 4 Discussion

A 10-year-old neutered male dog treated with intratumoral Stelfonta for a left hindlimb MCT ultimately developed progressive local necrosis with a circumferential cicatrix, systemic inflammatory shock, and pancreatitis, necessitating left hindlimb amputation. The adverse events described in this case highlight a more severe outcome than is typically reported following intratumoral injection of Stelfonta. This patient developed profound local necrosis, likely resulting from a tourniquet effect secondary to a circumferential cicatrix. It should be noted that initial superficial and subcutaneous necrosis and sloughing were the expected consequences of the Stelfonta injection.

The underlying etiology of the extensive necrosis and systemic effects (pancreatitis and shock) in this patient is likely multifactorial. The local effects are closely related to the mechanism of action of tigilanol tiglate. The drug induces rapid vascular disruption and oncolysis, processes that inherently cause local tissue destruction and trigger damage-associated molecular patterns (DAMPs) (3). As demonstrated in human research (8) and in this case, it is plausible that these inflammatory mediators propagated beyond the local site, triggering a systemic inflammatory cascade. Potential mast cell degranulation cannot be ignored and is also a plausible primary cause of the systemic disease. These pathways may have acted synergistically, further exacerbating vascular leakage and tissue swelling caused by Stelfonta and amplifying the subsequent systemic inflammatory response, thereby creating a cycle of local compromise and distant organ dysfunction.

The decision to continue prolonged wound management versus proceeding with early amputation represents a difficult clinical and ethical consideration. The manufacturer recommends minimal intervention in wound care. However, in this case, more than 7 weeks of intensive treatment were pursued prior to limb removal, during which the patient experienced persistent pain, infection, immobility, and recurrent hospitalization. Although amputation can be a definitive intervention that may prolong survival in some otherwise non-resectable neoplasms when performed early (9), it was not a preferred option for this owner early in the treatment process. Stelfonta was elected by the owner after the patient met the labeled criteria for appropriate case selection, including initial tumor size, location, and cytologic grade.

Early recognition of signs such as progressive necrosis, circumferential scarring, escalating pain, or distal ischemia should prompt immediate reassessment of the therapeutic approach. Amputation should be considered when wounds fail to heal within 4–6 weeks post-injection, infection persists despite appropriate antimicrobial therapy, or quality-of-life markers such as systemic compromise, refractory pain, or significant functional loss develop. Based on the findings of this report, monitoring for systemic inflammation and pancreatitis through clinical and laboratory assessments is likely a good idea, alongside the use of culture-directed antimicrobial therapy to ensure targeted infection control.

To the best of the authors’ knowledge, this is the first report of pancreatitis and systemic inflammatory shock temporally associated with tigilanol tiglate in a canine patient. Clinicians utilizing this medication should remain vigilant for the possibility of unusually severe local tissue reactions and systemic inflammatory complications and should consider early surgical intervention when objective criteria for limb compromise or systemic risk are met.

## 5 Conclusion

This case is unique in demonstrating the development of severe systemic illness, progressive distal extremity necrosis, and eventual limb amputation following a single intratumoral injection of Stelfonta. TT can induce severe local reactions and may also have the potential to cause systemic complications. Regardless of the underlying cause, this report emphasizes the principle that clinicians should closely monitor patients for systemic illness and wound complications, particularly in distal limb tumors where circumferential scarring can compromise perfusion.

## Data Availability

The original contributions presented in the study are included in the article/Supplementary material, further inquiries can be directed to the corresponding author/s.
