# Supplementary figures and images for: Case Report: Severe wound formation following intratumoral tigilanol tiglate treatment resulting in limb amputation in a 10-year-old male dog
Source: Front Vet Sci. 2026 Apr 16;13:1757258. doi: 10.3389/fvets.2026.1757258 (PMC13128416; doi:10.3389/fvets.2026.1757258)

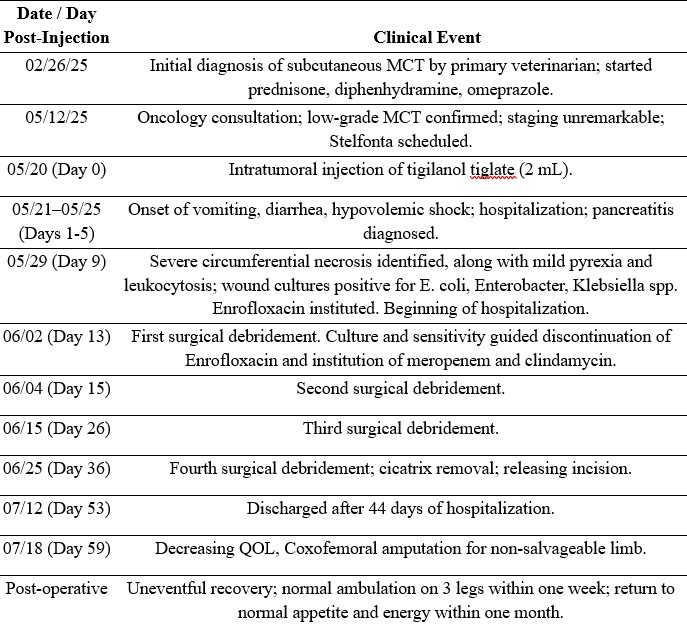

Supplement: Supplementary file 1 [file Image_1.JPEG]
